# Examining relationship between unhealthy lifestyle and life satisfaction among new students at Tehran University of Medical Sciences

**DOI:** 10.1371/journal.pone.0344179

**Published:** 2026-07-10

**Authors:** Mohamad Eshaghi, Keyvan Karimi, Nekoo Panahi, Yosra Azizpour, Farideh Razi, Anis Gharajeh, Samaneh Akbarpour, Fatemeh Hadavandsiri

**Affiliations:** 1 Non-Communicable Diseases Research Center, Endocrinology and Metabolism Population Sciences Institute, Tehran University of Medical Sciences, Tehran, Iran; 2 Occupational Sleep Research Center, Baharloo Hospital, Tehran University of Medical Sciences, Tehran, Iran; 3 Metabolic Disorders Research Center, Endocrinology and Metabolism Molecular-Cellular Sciences Institute, Tehran University of Medical Sciences, Tehran, Iran; 4 Clinical Research Development Unit, Shariati Hospital, Tehran University of Medical Sciences, Tehran, Iran; 5 Metabolomics and Genomics Research Center, Endocrinology and Metabolism Molecular-Cellular Sciences Institute, Tehran University of Medical Sciences, Tehran, Iran; 6 Endocrinology and Metabolism Research Center, Endocrinology and Metabolism Clinical Sciences Institute, Tehran University of Medical Sciences, Tehran, Iran; 7 Sleep Breathing Disorders Research Center (SBDRC), Tehran University of Medical Sciences, Tehran, Iran; Shahrood University of Medical Sciences, IRAN, ISLAMIC REPUBLIC OF

## Abstract

**Background:**

Transitioning to university is a critical period marked by lifestyle changes, academic pressures, and increasing autonomy, which can influence students’ overall well-being. Previous research suggests that unhealthy lifestyle behaviors—including poor diet, insufficient physical activity, inadequate sleep, and ineffective stress management—may negatively impact life satisfaction among young adults.

**Methods:**

This cross-sectional study examined the relationship between lifestyle and life satisfaction among newly enrolled undergraduate students at Tehran University of Medical Sciences in 2024. Participants completed structured questionnaires assessing multiple lifestyle domains (diet, physical activity, sleep, smoking, alcohol use) and life satisfaction using validated Iranian questionnaires. Lifestyle scores were categorized as “healthy” or “unhealthy,” and life satisfaction as “good” or “poor.” Associations were analyzed using Pearson’s chi-square test and multivariable logistic regression, adjusting for age, sex, socioeconomic status, and anxiety.

**Results:**

Overall, of the 419 students, 294 (70.2%) reported good life satisfaction. Socioeconomic status and anxiety levels differed significantly across life satisfaction groups (p < 0.001). Students with healthier dietary patterns reported higher life satisfaction (76.6% vs. 60.4%, p = 0.02). Multivariable analysis indicated that students with healthy lifestyles had significantly higher odds of good life satisfaction compared to those with unhealthy lifestyles (adjusted odds ratio [aOR = 1.91; 95% confidence interval [CI]: 1.10,3.35; p = 0.02). Each one-unit increase in lifestyle score was associated with a 28% increase in the odds of good life satisfaction (aOR = 1.28; 95% CI: 1.05,1.57; p = 0.02).

**Conclusion:**

Unhealthy lifestyle behaviors are negatively associated with life satisfaction among new university students. Interventions promoting balanced nutrition, regular physical activity, adequate sleep, and overall health-conscious behaviors may enhance students’ life satisfaction and well-being.

## Introduction

Lifestyle behaviors play a crucial role in shaping both physical and mental determinants of a wide range of healthcare outcomes [1]. Modifications of risk factors such as inadequate exercise levels, poor dietary practices, poor sleeping patterns, alcohol/drug abuse, and poor stress management play an important role in the global incidence of various chronic ailments [2,3]. Healthcare students, despite being comprehensively briefed on healthcare knowledge, tend to adopt unhealthy lifestyles owing to immense academic stress and work-related pressures [4,5]. About 60% of healthcare outcomes have been attributed to lifestyle factors [6,7]. Investigations carried out in Iran and the Asian continent have found that a substantial portion of healthcare students tend to have a poor to moderate lifestyle [8–11].

Life satisfaction is commonly defined as individuals’ subjective evaluation of their overall quality of life in relation to their personal standards and expectations, and is widely regarded as a core component of subjective quality of life and an important indicator of mental well-being [12]. Current evidence suggests that life satisfaction among medical students is adversely affected by chronic psychological stress inherent to medical training, with perceived stress accounting for approximately 12% of the variance in life satisfaction [13]. The mentioned students constitute a high-risk group in respect to mental well-being [14].

Previous research indicates that a lack of exercise, poor sleeping patterns, an unbalanced diet, and the misuse of substances correlate with reduced life satisfaction among university students [15,16]. In particular, there needs to be more information on such matters in Iranian medical students, studies among Iranian medical students reveal that physical activity constitutes the weakest dimension of health behaviors, with significantly low scores compared to other lifestyle factor [17].

We aim to explore how lifestyle behaviors are associated with life satisfaction in first-year medical students in Iran. This stage is important because students’ habits and lifestyles upon entering university often reflect their experiences before medical school, shaped by personal, social, and educational factors. Understanding this allows us to distinguish the influence of medical training from pre-existing patterns related to family, school, and society. By identifying these factors early, we seek to support students’ health and well-being and promote a balanced and meaningful medical education journey.

## Methods

### Study design and data collection process

This study is a cross-sectional study designed to investigate the relationship between lifestyle and life satisfaction in new students at Tehran University of Medical Sciences (TUMS) in 2024.

This study was a cross-sectional analysis of baseline (Phase 1) data derived from an ongoing three-year prospective cohort study designed to examine lifestyle characteristics and non-communicable disease (NCD) risk factors among first-year medical students at TUMS. Baseline data were collected between December 4, 2024, and February 19, 2025. The overall cohort aims to assess longitudinal changes in lifestyle and their associations with health status, NCDs, and academic performance over time. Phase 1 comprised three sequential steps, and the present analysis was based on data obtained from Step 1, in which structured questionnaires were administered. Participants completed questionnaires covering major lifestyle domains, including dietary habits, physical activity, sleep patterns, tobacco use, alcohol consumption, and overall life satisfaction [18].

The inclusion criteria for this study encompassed newly enrolled students at TUMS who were aged 18 years or older and had provided written informed consent for participation. Exclusion criteria included students originating from countries other than Iran. Additionally, an open invitation was disseminated to encourage voluntary participation from all interested individuals.

### Sample size

Of the 422 people who participated in this study, 419 responded to the life satisfaction questionnaire (outcome). In the current secondary analysis, we used pairwise deletion in the descriptive reports, and missing data for each variable were dropped. For the final regression models, we excluded all cases with missing data on the independent variables of interest in this study, resulting in a total of 344 participants analyzed.

### Variables definition

The present study utilized data collected through a comprehensive, multidimensional, standardized questionnaire that had been administered in the parent study to assess lifestyle factors and life satisfaction [18]. Demographic characteristics, including age, sex, marital status, place of residence, socioeconomic status (SES), field of study, and parental education, were collected. Tobacco use was assessed based on current use (past 12 months) and passive exposure, while alcohol consumption was evaluated as current use (yes/no). Dietary habits were scored according to adequate consumption of five major food groups (fruits, vegetables, proteins, grains, and dairy), regular meal patterns, breakfast consumption, and avoidance of sugar-sweetened beverages, fast food, and processed salty foods, and subsequently categorized into tertiles (unhealthy, partially healthy, and healthy). Physical activity was measured using the WHO STEPS physical activity module and categorized as inactive (0 minutes/week), insufficient (<150 minutes/week), or sufficient (≥150 minutes/week). Sleep quality was assessed as normal or abnormal. A composite lifestyle score (range: 0–8) was calculated by summing the scores for diet (0–2), physical activity (0–2), smoking status (0–2), alcohol use (0–1), and sleep quality (0–1). All instruments demonstrated established psychometric properties in Iranian populations. SES was assessed using a self-reported ordinal question in which participants classified their household socioeconomic position into five nationally referenced categories (upper, upper-middle, middle, lower-middle, and lower). For analytical purposes, these categories were recoded into three levels: upper and upper-middle were combined as high SES, middle was considered as average SES, and lower-middle and lower were grouped as low SES. This categorization approach is consistent with previously published studies using self-rated SES measures [19].

Physical activity was assessed using a modified version of the Global Physical Activity Questionnaire (GPAQ-v2) [20,21], adapted for students by excluding work-related items. Mental health status was evaluated using the 7-item Generalized Anxiety Disorder scale (GAD-7) [22], to assess anxiety-related symptoms [23,24]. Sleep quality and sleep-related disorders were examined using the Pittsburgh Sleep Quality Index (PSQI) [25–27].

Additionally, life satisfaction was assessed using the five-item Satisfaction with Life Scale (SWLS), in which participants indicated their level of agreement on a five-point Likert scale from strongly disagree to strongly agree [28–30]. All instruments employed in this study have been previously adapted and evaluated for use in Iranian populations.

### Data assurance and quality control

A fundamental aspect of the main study was the rigorous assurance and maintenance of data quality. Careful oversight was applied to all stages, including sampling, survey administration, data collection and entry, and analysis. To guarantee precise data capture and thorough questionnaire completion, the entire surveying process for each participant was systematically recorded within the study’s information system.

The monitoring team developed a detailed checklist to guide and evaluate both questionnaire completion and anthropometric measurements. This checklist covered preparation, questionnaire design, and critical procedural elements during data collection. Supervisors used this tool to track adherence to protocols and document any challenges encountered. Reports generated from these checklists were submitted daily to the project manager, enabling prompt identification and resolution of any issues to maintain the study’s data integrity [18].

### Statistical method

The level of life satisfaction and its distribution across categories of each independent variable were examined using Pearson’s chi-square test. The hypothesis was that |individuals with healthier lifestyle behaviors would have greater satisfaction than those with an unhealthy lifestyle pattern. The binary logistic regression model was used to examine the association between lifestyle and life satisfaction score. Odds ratios (ORs) were estimated and presented with 95% confidence intervals (CIs). The adjusted model controlled for age, sex, SES, and anxiety variables as potential confounders, and the adjusted odds ratio (aOR) for the relationship between lifestyle and life satisfaction was reported. The outcome or dependent variable of the analysis was the life satisfaction score, categorized as a binary factor. An overall life satisfaction score less than 20 was defined as ‘poor life satisfaction,’ and participants with a score greater than or equal to 20 were classified as having ‘good life satisfaction` [31]. Independent variables in the logistic regression were lifestyle scores, which were calculated based on lifestyle behaviors including physical activity, smoking habits, diet, alcohol consumption, and sleep duration. The total lifestyle score was categorized as less than 5 (unhealthy lifestyle) and greater than or equal to 5 (healthy lifestyle). All statistical procedures were performed using Stata version 17, and *p* < 0.05 was considered statistically significant in all analyses.

### Ethics approval and consent to participate

The study received ethical approval from the Research Ethics Committees of the Endocrine & Metabolism Research Institute at Tehran University of Medical Sciences (ID: IR.TUMS.EMRI.REC.1403.112). All participants provided written informed consent in accordance with the principles outlined in the Declaration of Helsinki. Participation was entirely voluntary, and individuals retained the right to withdraw from the study at any point without penalty.

## Results

The participants consisted of a total of 419 first-year university students from different fields, with a mean age of 19.41 years (standard deviation = 2.71). Table 1 contains the prevalence of life satisfaction by socio demographic and health related factors. Females constituted the majority of the study population, accounting for 225 participants (53.7%), compared to 194 males (46.3%). The prevalence of poor life satisfaction was 29.8% (125 participants), while the prevalence of good life satisfaction reached 70.2% (294 participants) among the study population. The prevalence of good life satisfaction was higher among students living at home compared with those residing in dormitories (71% vs. 69%). Students with higher SES also showed a notably greater prevalence of good life satisfaction (81.9%), whereas poor life satisfaction was most frequent among those with lower SES (52.7%). In addition, good life satisfaction was most common among individuals with low anxiety levels (81.9%), while the highest prevalence of poor life satisfaction was observed among those with moderate anxiety (49.3%). Among all variables examined, only SES and anxiety levels showed significant difference with life satisfaction (*p* < 0.001), while other variables were not significantly different.

**Table 1:** Prevalence of life satisfaction by socio-demographic and health-related factors.

Variable	Poor life satisfaction N (%)	Good life satisfaction N (%)	Total	*p* value
Total	125 (29.8%)	294 (70.2%)		
**Sex**				
Female	59 (26.2%)	166 (73.7%)	225	0.082
Male	66 (34%)	128 (65.9%)	194	
**Place of residence**				
Home	68 (28.9%)	167 (71%)	235	0.650
Dormitory	57 (30.9%)	127 (69%)	184	
**Socioeconomic status**				
Low	29 (52.7%)	26 (47.2%)	55	0.001<
Average	75 (30.2%)	173 (69.7%)	248	
High	21 (18.1%)	95 (81.9%)	116	
**Anxiety**				
Low	28 (18%)	127 (81.9%)	155	0.001<
Mild	56 (32.5%)	116 (67.4%)	172	
Moderate	36 (49.3%)	37 (50.6%)	73	

Missing data on Anxiety = 19.

Table 2 shows the prevalence of life satisfaction among participants according to their lifestyle behaviors.

**Table 2:** Prevalence of life satisfaction by lifestyles.

Variable	Poor life satisfaction N (%)	Good life satisfaction N (%)	Total	p value
**Diet**				
Unhealthy	55 (39.5%)	84 (60.4%)	139	0.019
Partially healthy	46 (26.7%)	126 (73.2%)	172	
Healthy	15 (23.4%)	49 (76.5%)	64	
**PA**				
Inactive	19 (40.4%)	28 (59.5%)	47	0.131
Insufficient	43 (32.5%)	89 (67.4%)	132	
Sufficient	59 (26.5%)	163 (73.4%)	222	
**Smoking**				
Current	10 (37%)	17 (62.9%)	27	0.286
Passive	72 (31.3%)	158 (68.7%)	230	
No	33 (24.8%)	100 (75.1%)	133	
**Alcohol**				
Yes	8 (44.4%)	10 (55.5%)	18	0.174
No	112 (29.4%)	269 (70.6%)	381	
**Sleep**				
Abnormal	79 (33.7%)	155 (66.2%)	234	
Normal	39 (25.4%)	114 (74.5%)	153	0.084

Missing data on each variable: Diet = 44, Physical Activity (PA) =28, Smoking = 29, Alcohol = 20, Sleep = 32

The highest prevalence of good life satisfaction was observed among students with a healthy diet (76.5%), adequate physical activity (73.4%), non-smoking status (75.1%), no alcohol consumption (70.6%), and normal sleep duration (74.5%). In contrast, poor life satisfaction was most prevalent among those with an unhealthy diet (39.5%), no physical activity (40.4%), current smoking (37%), alcohol use (44.4%), and abnormal sleep patterns (33.7%). Additionally, former smokers showed a higher prevalence of good life satisfaction (68.7%) compared with current smokers (62.9%). Dietary pattern was the only variable that showed a statistically significant difference between groups (*p* = 0.02), while no significant differences were observed for the other lifestyle factors.

Table 3 presents the results of multivariable logistic regression analysis examining the association between lifestyle and life satisfaction. After adjusting for age, sex, socioeconomic status, and anxiety levels, students with healthy lifestyles had significantly higher odds of reporting good life satisfaction compared to those with unhealthy lifestyles (aOR = 1.91, 95% CI: 1.10,3.35; *p* = 0.02). Additionally, each one-unit increase in lifestyle score was associated with a 28% increase in the odds of good life satisfaction (aOR = 1.28, 95% CI: 1.05,1.57; *p* = 0.02).

**Table 3:** The association of a healthy lifestyle with life satisfaction in medical students.

Independent variable	Crude OR (95%CI)	*p* value	Adjusted OR (95%CI)	*p* value
Healthy lifestyle (vs. unhealthy lifestyle)	2.19 (1.29,3.70)	0.003	1.91 (1.10,3.35)	0.022
Lifestyle score (0–8)	1.39 (1.15, 1.69)	0.001	1.28 (1.05, 1.57)	0.019

Abbreviations: OR: Odds Ratio; CI: Confidence Interval, n = 344 (complete-case analysis).

*Adjusted for Age, Sex, SES, and Anxiety in logistic regression model.

## Discussion

The aim of this cross-sectional study was to examine the association between lifestyle habits and life satisfaction among first-year students at TUMS. The findings demonstrated a positive and statistically significant association between healthier lifestyle habits and higher life satisfaction, which remained robust after adjustment for age, gender, socioeconomic status, and anxiety level in multiple regression analyses. Students with healthier habits demonstrated higher odds of good life satisfaction compared with their less healthy counterparts. Among the individual lifestyle components assessed, dietary habits were the only factor significantly associated with life satisfaction.

Our findings highlight the important role of nutrition in shaping life satisfaction among first-year university students, consistent with observations by Hong et al. [32] in 15-year-old adolescents. Despite the age difference, dietary behaviors in both groups appear similar, supporting the relevance of this comparison. Unhealthy habits—such as frequent consumption of soft drinks, fast foods, and snacks—were linked to lower life satisfaction and higher symptoms of depression. Moreover, other unfavorable lifestyle behaviors, including inadequate sleep, high screen time, and low physical activity, have been shown to negatively affect quality of life and life satisfaction in university populations [33]. These patterns emphasize that lifestyle choices impact not only physical health but overall well-being. Our results reinforce broader epidemiological evidence that maintaining healthy habits across multiple domains can enhance life satisfaction and underscore the importance of promoting comprehensive health interventions among young adults entering university.

Our findings showed that students with lower levels of anxiety reported higher life satisfaction. This is in line with previous research in university students, where higher anxiety levels were generally associated with lower life satisfaction [34,35]. These studies collectively highlight the relevance of anxiety for well-being in student populations and support the importance of addressing anxiety to promote life satisfaction and overall mental health.

Our finding that students with healthier lifestyles report higher life satisfaction aligns with international evidence showing positive associations between healthy behaviors (particularly physical activity, adequate sleep and stress management) and life satisfaction among university students [36,37]. This convergence across studies suggests that a balanced and health-conscious lifestyle supports both physical and psychological well-being during the university years. As this period often involves academic pressure and increasing autonomy, students who maintain regular physical activity, adequate sleep, and effective stress-management generally experience higher life satisfaction. Such behaviors can also enhance academic performance, social relationships, and self-efficacy, contributing to a greater overall sense of fulfillment.

Our study highlights that the lifestyle habits observed among first-year university students—formed largely under the influence of family, schooling, and broader social environments prior to entering university—are already linked to their reported life satisfaction. This suggests that the transitional period at the start of university may represent a critical window during which promoting healthier routines could have lasting benefits throughout students’ academic and professional lives. At the same time, the notable socioeconomic differences observed in life satisfaction emphasize the need for universities to provide equitable support by ensuring accessible nutritious food options, opportunities for physical activity, and adequate resources for stress management. Nevertheless, several limitations must be acknowledged. The cross-sectional design prevents causal inference, and the use of self-reported measures introduces the possibility of bias. Additionally, the study’s focus on a single medical university in Tehran limits generalizability to other regions or educational contexts. Although we adjusted for key confounders, factors such as prior mental health status, personality traits, academic expectations, and family dynamics may still influence the observed associations. Future research should adopt longitudinal designs to track changes across medical training and explore potential mediators and moderators that could clarify how lifestyle behaviors influence life satisfaction over time.

## Conclusions

This study shows a strong relationship between healthier lifestyle choices and better life satisfaction among first-year medical students at Tehran University of Medical Sciences. Students who followed healthier habits were almost twice as likely to report good life satisfaction. This was true regardless of demographic and psychosocial factors. The findings highlight how important lifestyle choices, especially diet, are for life satisfaction during the tough transition into medical training. They provide new insights into how lifestyle affects subjective satisfaction in a particularly stressed group. This underscores the need for focused, evidence-based efforts to encourage sustainable healthy behaviors in medical education. Institutions should create supportive environments and address economic inequalities to improve student health outcomes.
